# Surgical Treatment and Clinical Outcome of Nonfunctional Pancreatic Neuroendocrine Tumors: A 14-Year Experience From One Single Center

**DOI:** 10.1097/MD.0000000000000094

**Published:** 2014-11-07

**Authors:** Min Yang, Lin Zeng, Yi Zhang, An-ping Su, Peng-ju Yue, Bo-le Tian

**Affiliations:** Department of Hepato-bilio-pancreatic Surgery (MY, YZ, AS, PY, BT); and General Ward of Sports Medicine and Cardiopulmonary Rehabilitation (LZ), West China Hospital, Sichuan University, Chengdu, Sichuan Province, The People’s Republic of China.

## Abstract

Our primary aim of the present study was to analyze the clinical characteristics and surgical outcome of nonfunctional pancreatic neuroendocrine tumors (non-F-P-NETs), with an emphasis on evaluating the prognostic value of the newly updated 2010 grading classification of the World Health Organization (WHO).

Data of 55 consecutive patients who were surgically treated and pathologically diagnosed as non-F-P-NETs in our single institution from January 2000 to December 2013 were retrospectively collected.

This entirety comprised of 55 patients (31 males and 24 females), with a mean age of 51.24 ± 12.95 years. Manifestations of non-F-P-NETs were nonspecific. Distal pancreatectomy, pancreaticoduodenectomy, and local resection of pancreatic tumor were the most frequent surgical procedures, while pancreatic fistula was the most common but acceptable complication (30.3%). The overall 5-year survival rate of this entire cohort was 41.0%, with a median survival time of 60.4 months. Patients who underwent R0 resections obtained a better survival than those who did not (*P *< 0.005). As for the prognostic analysis, tumor size and lymph invasion were only statistically significant in univariate analysis (*P* = 0.046 and *P *< 0.05, respectively), whereas the newly updated 2010 grading classification of WHO (G1 and G2 vs G3), distant metastasis, and surgical margin were all meaningful in both univariate and multivariate analysis (*P* = 0.045, 0.001, and 0.042, respectively).

Non-F-P-NETs are a kind of rare neoplasm, with mostly indolent malignancy. Patients with non-F-P-NETs could benefit from the radical resections. The new WHO criteria, distant metastasis and surgical margin, might be independent predictors for the prognosis of non-F-P-NETs.

## INTRODUCTION

With an annual incidence of <5 per 1,000,000, pancreatic neuroendocrine tumors (p-NETs) are not common, although they showed an increasing tendency in recent decades.^1–3^ Consisting of a group of heterogeneous tumors, p-NETs account for only 1% to 2% of all pancreatic tumors.^4^ We had a common and simple practice to label p-NETs as functional if patients present the symptoms related to hormone overproduction and nonfunctional if they do not.^5^

Accounting for 35% to 50% of all p-NETs, nonfunctional pancreatic neuroendocrine tumors (non-F-P-NETs) were rare and mostly occurred in the fourth or fifth decade of life.^6–8^ Non-F-P-NETs may react positive for some endocrine hormone in the immunohistochemical staining, as well as get some rise of a certain level of hormone in blood, but they would not lead to the typical clinical manifestations of hormone overproduction for the lack of enough hormonal concentrations.^9^ Tumors often had grown to an advanced stage when patients were admitted into hospital, because of the nonspecific symptoms that were usually difficult to be discovered in the early days, such as nausea and vomiting, abdominal pain and distension, abdominal mass, and others. It was reported that over 60% of non-F-P-NETs were malignant when first diagnosed. Meanwhile, probably because of the indolent biological behaviors, patients with non-F-P-NETs often obtained much better survival than those with pancreatic adenocarcinoma.^10,11^

To the best of our knowledge, studies of non-F-P-NETs concentrated on evaluating the surgical outcome and prognosis were not common because of their heterogeneity and rarity, especially using the newly updated 2010 grading classifications of the World Health Organization (WHO)^12^ and the tumor node metastasis (TNM) staging system of the European Neuroendocrine Tumor Society (ENETS).^13^ In our opinion, with an obviously increasing tendency of this disease in recent decades, necessities were needed to do some new researches on non-F-P-NETs. In the present study, we reviewed the 14-year experience in our single institution of 55 consecutive resections with non-F-P-NETs to analyze the clinical characteristics of non-F-P-NETs, assess their long-term survival following surgical treatments, and discuss their prognosis, with an emphasis on evaluating the prognostic value of the new WHO criteria.

## MATERIALS AND METHODS

### Patient Selection

Fifty-five patients who were all surgically treated and pathologically diagnosed as non-F-P-NETs in West China Hospital, Sichuan University, Chengdu, The People’s Republic of China, from January 2000 to December 2013 were enrolled in our study, while patients with only preoperatively clinical or imaging suspicions but not postoperatively pathological confirmations of non-F-P-NETs were excluded. This research was approved by the local ethics committee, and written consent was provided for patient information to be used for research purposes. Data, including patients’ demographics (gender and age), clinical presentations at the time of admission, surgical data (procedures and duration of operation, intraoperative findings, etc.), postoperative morbidity and mortality rates, were all retrospectively reviewed from patients’ medical records. We excluded few patients who received chemotherapy or other treatments after resection.

### Tumor Characteristics

Tumors that were pathologically diagnosed as pancreatic neuroendocrine tumors (p-NETs) but without recognizable and typical syndromes related to hormone overproduction were defined as non-F-P-NETs in our study. Features of the tumors (size, location, lymph invasion and distant metastasis, surgical margin, mitotic count, Ki-67, etc) were all based on intraoperative findings and pathological analysis. The newly updated WHO 2010 grading classifications and the ENETS 2006 TNM staging systems were both applied whenever possible. For some potentially missing pathological data of patients before 2010, we redid the hematoxylin and eosin staining and immunohistochemical analysis of the excised formalin-fixed and paraffin-embedded specimen. The WHO new criteria were quoted as follows: NET G1 (neuroendocrine tumor G1: mitotic count ≤2/10HPF, Ki-67 ≤2%); NET G2 (neuroendocrine tumor G2: mitotic count 2–20/10HPF, Ki-67 3–20%); NEC G3 (neuroendocrine carcinoma G3: mitotic count >20/10HPF, Ki-67 >20%).

### Postoperative Complications

Postoperative mortality was defined as death occurring in the first 30 postoperative days or prior to discharge from the hospital. Pancreatic fistula was mainly classified according to the International Study Group on Pancreatic Fistula definition^14^and defined as drainage of any measurable volume of amylase-rich fluid, at least 3 times the upper normal limit of serum amylase concentration on or after the third postoperative day. Delayed gastric emptying, intra-abdominal infection, wound infection, and other complications were all defined by the standard accepted criteria.

### Follow-Up and Survival

Follow-up was done by telephone, office visit, or outpatient clinic during April and May, 2014. Five patients were lost to follow-up and were excluded from the survival analysis. Overall survival was calculated as the number of months from the date of operation to the day of last follow-up visit or time of death. Also, we excluded few patients who died of other causes when selecting the experimental subjects.

### Statistical Analyses

Data were presented as mean ± standard error of mean unless otherwise indicated. Differences in the continuous quantitative variables of demographics, operative data were analyzed using analysis of variance. Kaplan–Meier estimates of survival were plotted, and survival differences were analyzed using the log-rank test. Univariate and multivariate analyses were used to explore the effects of several prognostic factors by the cox regression proportional hazards model. Differences with two-sided *P* value <0.05 was considered statistically significant. Statistical analyses were performed using IBM SPSS17.0 statistical software.

## RESULTS

### Demographics and Manifestations

A total of 55 consecutive patients with non-F-P-NETs between January 2000 and December 2013 underwent pancreatic surgery in West China Hospital, Sichuan University (Table 1). The present cohort was comprised of 31 males and 24 females, with a mean age of 51.24 ± 12.95 years and a median of 54 (ranging from 14 to 75). Tumors diameter ranged from 1 to 10 cm, with an average of 4.85 ± 2.55 cm and a median of 5. Twenty-eight tumors were located in the head (including 5 cases in the uncinate), 15 in the body, and 12 in the tail. There were respectively 5, 18, 20, and 12 cases from stage T1 to T4. Twelve patients were pathologically confirmed to have invasions of lymph node, whereas 10 patients had distant metastases. In terms of the TNM staging system of the ENETS in 2006, stages I, II, II, and IV were defined in 5, 33, 7, and 10 patients, respectively. The new WHO 2010 grading classifications was possible for all patients with a distribution of 15, 32, and 8 in NET G1, NET G2, and NEC G3, respectively. Patients with non-F-P-NETs often presented nonspecific manifestations (Table 2).

**TABLE 1:**
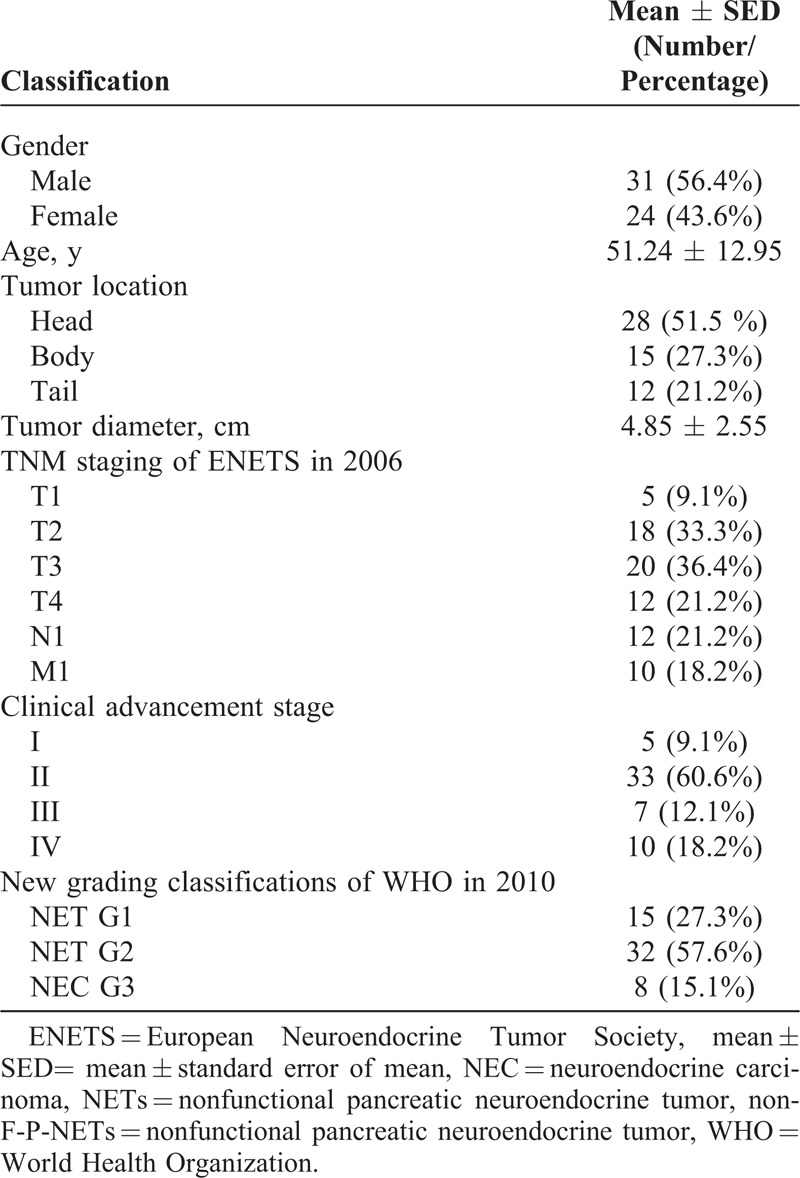
Clinical Data of the 55 Patients With Non-F-P-NETs

**TABLE 2:**
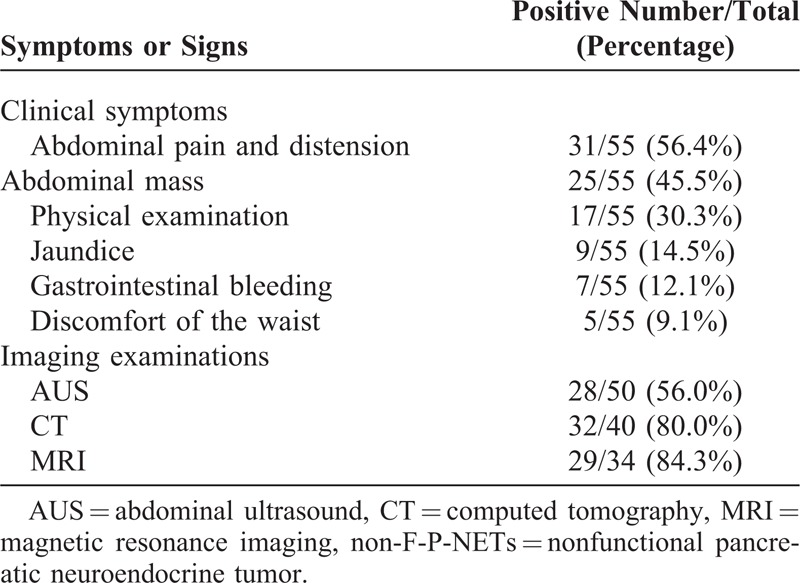
Clinical Manifestations and Positive Imaging Examinations of Non-F-P-NETs

### Operations and Complications

Pancreatic operations were performed for all patients in this study (Table 3). A total of 50 patients underwent curative resections (R0 resection, 90.9%), in which the distal pancreatectomy was the most common procedure (36.4%), followed by pancreaticoduodenectomy and local resection of pancreatic tumor (27.3% and 21.2%, respectively). Palliative operation (not R0 resection, 9.1%) was carried out for only 5 patients, where the biopsy of pancreatic mass and the implantation of radioactive iodine particles were both simultaneously performed. The operation duration ranged from 100 to 510 minutes, with an average of 239.62 ± 110.23 minutes. The overall postoperative duration of hospitalization and total duration of hospitalization ranged from 5 to 43 days and from 9 to 50 days, with a mean time of 14.01 ± 8.53 days and 20.86 ± 9.02 days, respectively. No notable differences were seen in age, tumor size, postoperative duration of hospitalization, and total duration of hospitalization among these surgical procedures (*P* = 0.677, 0.349, 0.541, and 0.557, respectively), while that was statistically significant in operation duration (*P* = 0.001) (Table 4).

**TABLE 3:**
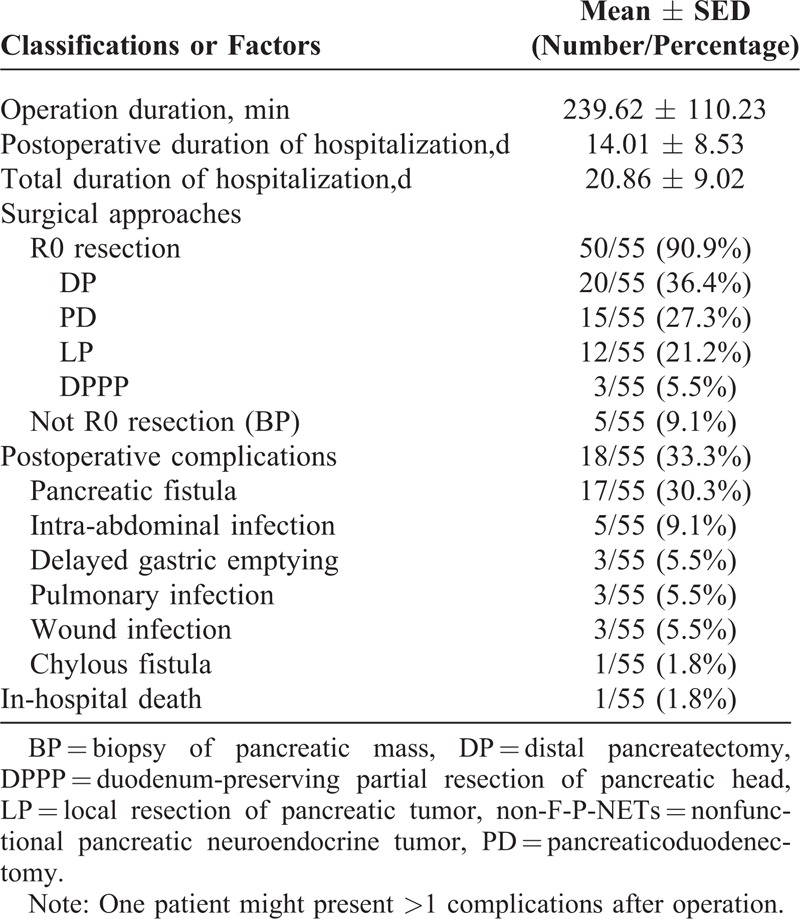
Surgical Characteristics of the 55 Patients With Non-F-P-NETs

**TABLE 4:**
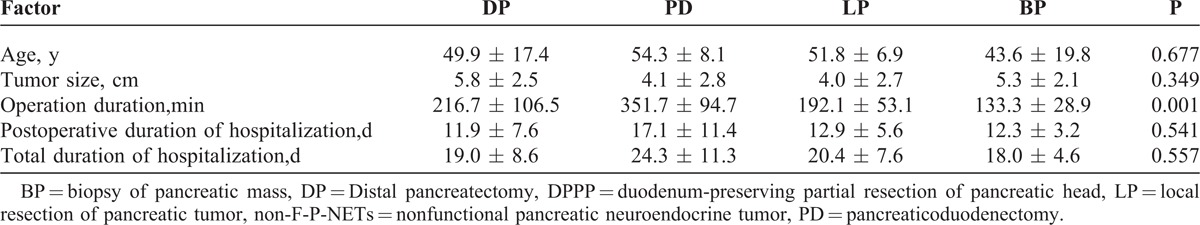
Comparison of the Main Surgical Procedures for Patients With Non-F-P-NETs

Postoperative complications occurred in 18 patients with an incidence of 33.3%, the most common of which was pancreatic fistula (Table 3). Although the morbidity of pancreatic fistula was a little high (30.3%), it was still acceptable for all of them either Type A or B, patients recovered well with proper medical treatments and unobstructed drainages. Other uncommon complications could also be well treated through appropriate conservative therapies. There were 2 reoperations because of the postoperative wound infection and only 1 in-hospital death because of the hypovolemic shock caused by intra-abdominal hemorrhage.

### Survival and Outcome

Follow-up was achieved in 50 patients, ranging from 3.6 to 154.2 months, with a median of 56.8 months. Five patients were lost to follow up and were excluded in the survival analysis (9.1%). Thirty-five patients (70.0%) were still alive till follow-up, whereas 15 patients (30.0%) have been dead because of the progression of diseases. Survival time of the entire cohort ranged from 3.7 to 137.6 months, with an average of 48.2 ± 28.9 months.

**FIGURE 1:**
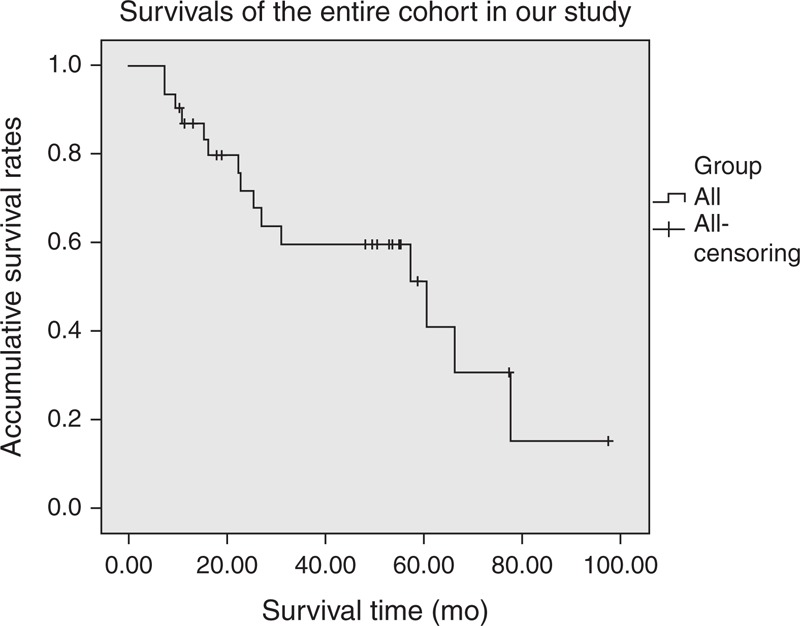
Overall 5-year survival rate of patients with non-F-P-NETs who underwent an operation was 41.0%, with a median overall survival time of 60.4 months (95% confidence interval, 20.9–99.8 months).

**FIGURE 2:**
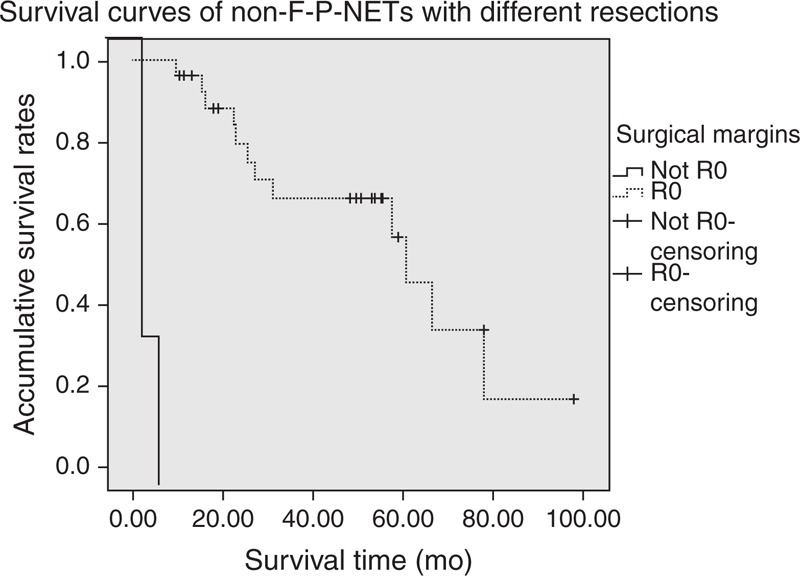
Comparison of survival for non-F-P-NETs with different resections. Survival time of patients who underwent R0 resection was statistically longer than that of patients who did not, whose median survival time was 60.43 and 7.40 months, respectively (*P* < 0.001).

**FIGURE 3:**
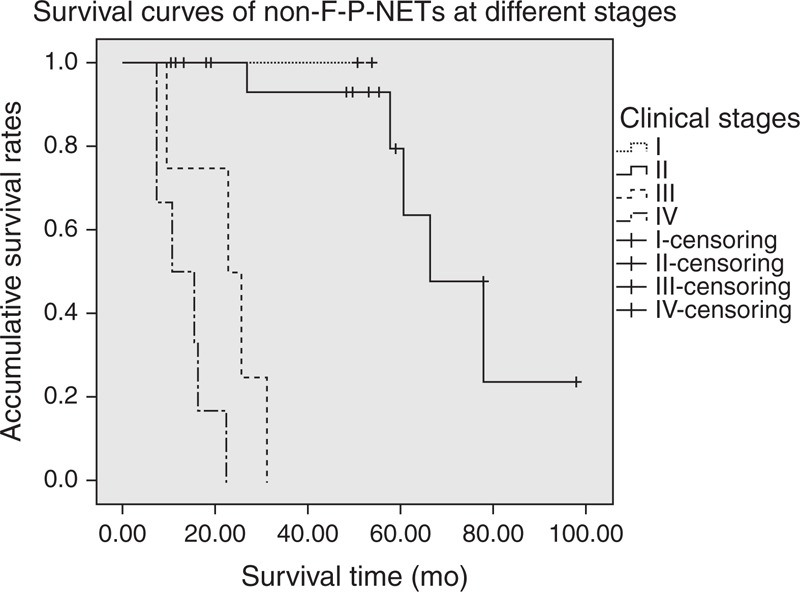
Comparison of survival for p-NETs in different stage. Survival time of patients in stage I was longer than those in stages III and IV (*P* = 0.049 and 0.034, respectively), while no notable differences were found between stages I and II (*P* = 0.705). Also, patients in stage II obtained better survival than those in stages III and IV (*P *< 0.005 and *P* < 0.005, respectively), as well as that between stages III and IV (*P* = 0.042).

**FIGURE 4:**
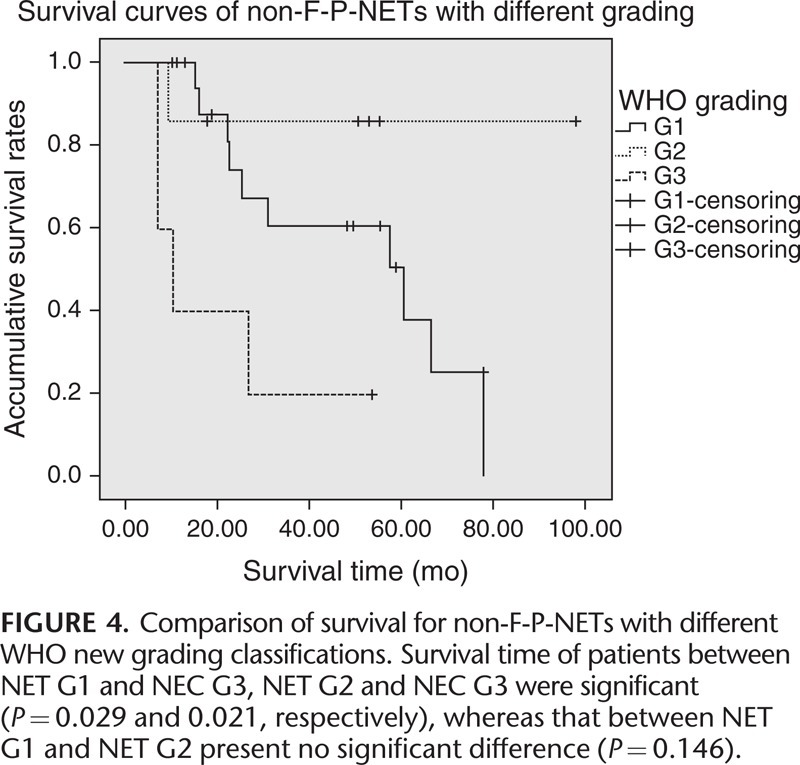
Comparison of survival for non-F-P-NETs with different WHO new grading classifications. Survival time of patients between NET G1 and NEC G3, NET G2 and NEC G3 were significant (*P* = 0.029 and 0.021, respectively), whereas that between NET G1 and NET G2 present no significant difference (*P* = 0.146).

Kaplan–Meier survival analysis concluded that the median survival time of our entire cohort was 60.4 months (95% confidence interval, 20.9–99.8 months), with a 5-year survival rate of 41.0% (Figure 1). Patients who underwent R0 resections showed a statistically better survival than those who did not (*P *< 0.005) (Figure 2). As for the TNM stage system of the ENETS (Figure 3), the survival time of patients in stage I was statistically longer than those in stages II and IV (*P* = 0.049 and 0.034, respectively), while no notable differences were found between stages I and II (*P* = 0.705). Also, patients in stage II obtained statistically better survival than those in stages III and IV (*P *< 0.005 and *P* < 0.005, respectively), as well as that between stages III and IV (*P* = 0.042). In terms of the updated WHO 2010 grading classifications (Figure 4), survival time of patients between NET G1 and NEC G3, NET G2 and NEC G3 were statistically significant (*P* = 0.029 and 0.021, respectively), whereas that between NET G1 and NET G2 present no significant difference (*P* = 0.146).

Univariate and multivariate analysis by cox regression model were performed to discuss the potential predictors of non-F-P-NETs. It could be realized in Table 5 that gender and age got no notable differences on the prognosis of non-F-P-NETs (*P* = 0.613 and 0.602, respectively), whereas those of tumor size, lymph invasion, distant metastasis, surgical margin, and the new WHO 2010 grading classifications were statistically significant in the univariate analysis (*P *> 0.05). Bringing the significant factors directly into multivariate analysis, we concluded that the new WHO criteria, distant metastasis, and surgical margin might be independent predictors of non-F-P-NETs (*P* = 0.045, 0.001, and 0.042, respectively).

**TABLE 5:**
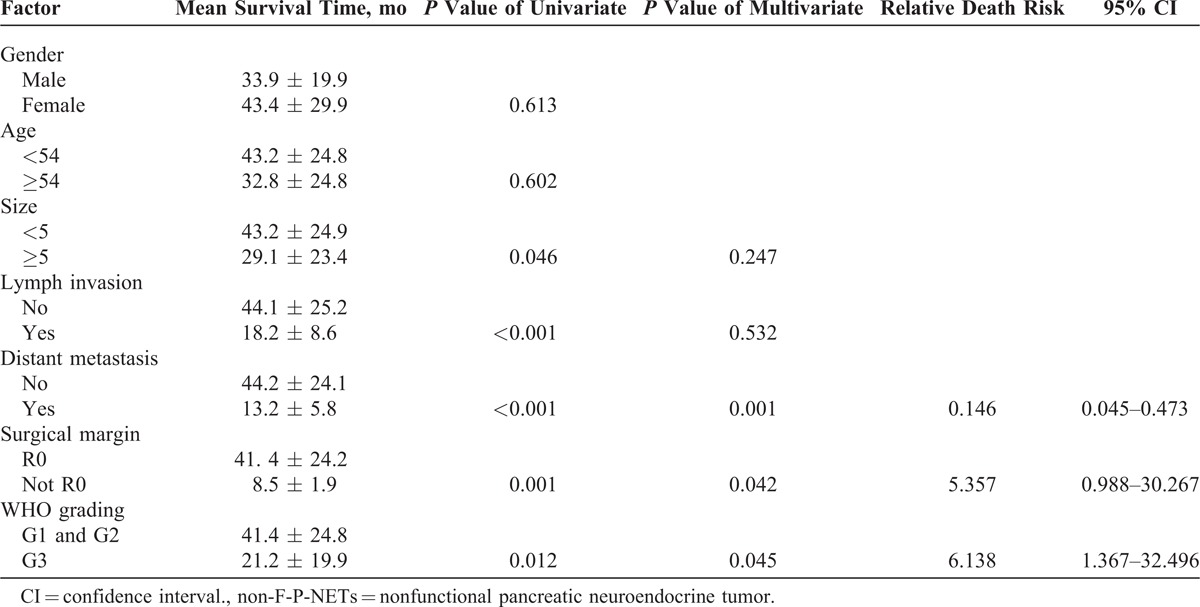
Univariate and Multivariate Analysis of the Possible Factors Influencing the Prognosis of Patients With Non-F-P-NETs

## DISCUSSION

Non-F-P-NETs are uncommon, while a large population-based study revealed that the annual incidence of non-F-P-NETs has increased from 1.4 to 3.0 per 1,000,000 from 1973 to 2004.^15^ Their diagnosis tends to be delayed because of the lack of typical hormone-related symptoms. Clinical manifestations of non-F-P-NETs were nonspecific. Tumors may lead to abdominal pain, abdominal mass, waist discomfort, as well as obstructive jaundice for the oppression of bile duct by tumors, or even nothing. In our study, 30.3% patients were asymptomatic (17/55); their placeholder lesions of pancreas were just accidentally detected by physical examinations. Imaging examinations contributed to locating the tumors, which had become the main methods of preoperative diagnosis. In our study, abdominal ultrasound, computed tomography (CT), and magnetic resonance imaging (MRI) were the most widely used, in which CT and MRI both showed a relatively high positive rate (80.0% and 84.3%, respectively).

Surgery is the main and only potentially curative treatment of non-F-P-NETs, with a basic principle of radical resection.^16,17^ Related studies by Hill et al^18^ and Gullo et al^8^ concluded that differences between patients with non-F-P-NETs who underwent operations and those who did not were significantly notable, while the research by Dralle et al^17^ indicated that patients with radical resections often obtained a statistically longer survival than those who underwent palliative operations. Evans et al^19^ also demonstrated that radical resections of non-F-P-NETs improved long-term survival. In the present study, the median survival time of patients who underwent R0 resections was 60.4 months, compared to 7.4 months of patients who did not (*P *< 0.005), which was in agreement with the previous reports. On the contrary, radical resections or palliative operations should be strived for as far as possible for patients with p-NETs although he/she manifest presentations of distant metastasis, for surgery might relieve patient’s symptoms, and reduce his/her subsequent treatments concerned with these diseases.^20,21^ However, their studies consisted of so many cases with functioning p-NETs that patients mostly present typical syndromes of hormone over-production. There are no available data or related reports to support debulking procedures for patients with unresectable non-F-P-NETs unless patients present the symptoms of obstruction.

The operative approaches differed from tumor size, location, and its relationship with surrounding tissues. Because of the preferred distributions of pancreatic head and the first diagnosed size of >2 cm, distal pancreatectomy and pancreaticoduodenectomy have been performed clinically on the rise.^22^ In our study, distal pancreatectomy was the main surgical procedure for patients who underwent R0 resection (36.4%), followed by pancreaticoduodenectomy (27.3%) and local resection of pancreatic tumor (21.2%). What is more, differences of age, size, and postoperative and total in-hospital durations among these main surgical procedures were not significant (*P *> 0.05), while that of operation duration was statistically notable (*P* = 0.001).

Because of the biologic rarity of non-F-P-NETs, it is often difficult to predict the prognosis for patients who underwent an operation, which is yet uncompleted and unclear.^23–25^ Based on a contemporary series of 163 patients, Solorzano et al^25^ concluded that tumor size was not a predictive factor for patients with non-F-P-NETs, which was in agreement with the 2 large-population-based studies by Bilimoria et al^26^ and Franko et al.^15^ However, 2 another high-quantity researches suggested that small tumors (<2–3 cm) were associated with better survival.^8,24^ In our study, tumors >5 cm (a critical value we redefined) present a statistically reduced long-term survival than those <5 cm in univariate analysis (*P* = 0.046). Therefore, it was probably reasonable to speculate that the larger size the tumor has, the bad prognosis the patients might acquire.

Another factor that could not reach a consensus was lymph invasion. Some studies found no survival predictive value of nodal metastases.^15,25,27,28^ In agreement with our conclusions (*P* = 0.532), Bahra et al^29^ reported that lymph node metastases did not seem to be significant in determining the survival of non-F-P-NETs, for their multivariate analysis of nodal stage showed no notable differences with respect to the estimated cumulative survival probability (*P* = 0.81). On the contrary, Bettini et al^24^ and Rindi et al^13^ both identified nodal metastases as a negative predictor of survival. So, combining with the results of our research, we speculated that lymph invasion might have some influence on prognosis of non-F-P-NETs, but could not probably be an independent predictor with much significance.

The value of Ki-67 positive rate for the prognosis of non-F-P-NETs has ever been confirmed. Bahra et al^29^ also concluded in 2006 that patients with Ki-67 over 2% showed a significantly decreased survival rate in multivariate analysis (*P* = 0.023), which was similar to the results by Rosa et al.^30^ A recent study in 2008 against the WHO classification systems evaluated 180 patients with non-F-P-NETs, which demonstrated poor differentiation as a negative predictor.^24^ However, researchers above had utilized either the solo Ki-67 or the old WHO grading classifications^31^ as the probable variables. In the present study, we evaluated the surgical outcome of non-F-P-NETs with the newly updated WHO 2010 grading system and discussed its prognostic value, in which we concluded that patients with non-F-P-NETs of NET G1 and NEC G2 gained statistically better survival rates than those of NEC G3 (*P* = 0.029 and 0.021, respectively), whereas survival between NET G1 and NET G2 present no notable difference (*P* = 0.146). Moreover, we demonstrated that the new WHO criteria might be an independent predictor both in the univariate and multivariate analysis (NET G1 and NEC G2 vs NEC G3: *P* = 0.012 and 0.045, respectively)

In addition, Bettini et al^24^ also demonstrated that distant metastatic spread as a negative prognostic marker. Franko et al^15^ reported that the median survival time of patients without distant metastasis was significantly longer than that of patients who had (8.4 and 1.0 years, respectively; *P *< 0.001). Bahra et al^29^ concluded that tumor-free resection margins were important and radical surgical procedures were justified in selected patients. Clearly listed in Table 5 in our study, besides the new WHO criteria, distant metastasis and surgical margin were also statistically significant along with independent prognostic factors of non-F-P-NETs, which meant that radical resection should be taken into consideration first before patients present the manifestations related to distant metastasis. On the contrary, Denecke et al^32^ evaluated radiological prognostic factors of hepatic metastases in patients with non-F-P-NETs from another perspective, in which they concluded that hypovascularization of liver metastases from G1 and G2 non-F-P-NETs reflected by hypoenhancement during the early contrast phases seemed to be associated with early tumor progression within 12 months (*P* = 0.039), and that patients of non-F-P-NETs with hypoenhancing metastases in liver should repeat biopsy for reassessment of grading so that early initiation of therapy could be considered as soon as possible.

## CONCLUSION

Non-F-P-NETs are rare but had the potential to be slow-growing malignancy associated with an expected survival. Surgery remains to play an important role in achieving a probably curative treatment. Our data confirmed that patients with non-F-P-NETs could benefit from the radical resections. Tumor size and lymph invasion might have some impacts on the prognosis of non-F-P-NETs, but they were only statistically significant in univariate analysis. Nevertheless, the newly updated grading classifications of WHO in 2010 present its statistical value in both univariate and multivariate analysis, which meant it might be an independent predictor, as well as the factors of distant metastasis and surgical margin.
