# Surgical Treatment and Clinical Outcome of Nonfunctional Pancreatic Neuroendocrine Tumors: A 14-Year Experience From One Single Center: Erratum

**DOI:** 10.1097/01.md.0000481351.78602.1f

**Published:** 2016-03-03

In the article “Surgical Treatment and Clinical Outcome of Nonfunctional Pancreatic Neuroendocrine Tumors: A 14-Year Experience From One Single Center”,^1^ which appeared in Volume 93, Issue 22 of *Medicine*, the co-first authorship line was incorrect. Drs. Yang, Zeng, and Zhang share first authorship. The article has since been corrected online.
